# Continuous, quantifiable, and simple osmotic preconcentration and sensing within microfluidic devices

**DOI:** 10.1371/journal.pone.0210286

**Published:** 2019-01-16

**Authors:** Andrew Jajack, Isaac Stamper, Eliot Gomez, Michael Brothers, Gavi Begtrup, Jason Heikenfeld

**Affiliations:** 1 Department of Biomedical Engineering, University of Cincinnati, Cincinnati, Ohio, United States of America; 2 Department of Electrical Engineering and Computing Systems, University of Cincinnati, Cincinnati, Ohio, United States of America; 3 Eccrine Systems, Incorporated, Cincinnati, Ohio, United States of America; University of Hong Kong, CHINA

## Abstract

Insurmountable detection challenges will impede the development of many of the next-generation of lab-on-a-chip devices (e.g., point-of-care and real-time health monitors). Here we present the first membrane-based, microfluidic sample preconcentration method that is continuous, quantifiable, simple, and capable of working with any analyte. Forward osmosis rapidly concentrates analytes by removing water from a stream of sample fluid. 10-100X preconcentration is possible in mere minutes. This requires careful selection of the semi-permeable membrane and draw molecule; therefore, the osmosis performance of several classes of membranes and draw molecules were systematically optimized. Proof-of-concept preconcentration devices were characterized based on their concentration ability and fouling resistance. *In-silico* theoretical modeling predicts the experimental findings and provides an engineering toolkit for future designs. With this toolkit, inexpensive ready-for-manufacturing prototypes were also developed. These devices provide broad-spectrum detection improvements across many analytes and sensing modalities, enabling next-generation lab-on-a-chip devices.

## Introduction

Advances in microfluidics have driven the miniaturization, integration, and automation of traditional analytical techniques for measuring biologically-relevant molecules. Lab-on-a-chip devices have targeted high-impact applications, such as point-of-care diagnostics [1,2], but new frontiers, such as real-time health monitoring [3], remain untapped. In many cases, the physiological concentrations of analytes found in non-invasively accessible biofluids (e.g., sweat, saliva, tears) are orders of magnitude below the range of detection of available analyte sensors [4] or have binding and dissociation kinetics that cause equilibrium to take hours to achieve [5,6].

One possible approach is to improve the range of detection of analyte sensors. To do this, the sensitivity (signal magnitude per unit concentration) must increase, and the limit of detection (a function of signal-to-noise ratio) must decrease. Unfortunately, no universal method exists to downshift the range of detection for all sensors. Downshifting the range of detection either requires the tedious development of new probe chemistries (antibodies, aptamers, etc.) repeated for each analyte of interest or enhancement of the signal produced from a single binding event. Multiple options exist for improving the latter; the most advanced sensor transducers, such as analyte probes on electronic graphene [7], optically resonant surface plasmon [8], or mechanically resonant micro-cantilevers [9], downshift the range of detection by increasing sensitivity rather than improving the binding interaction between the analytes and the probes. However, in all of these cases, enhancing the signal also enhances the noise, which is significant not only in complex biological media but also in electronic sensing.

An alternative approach is to concentrate the sample before sensing. By bringing analytes into the detection range of sensors, concentration techniques can improve detection regardless of the sensing modality. For this reason, preconcentration methods (e.g., liquid-liquid extraction, solid phase extraction, crystallization, or precipitation) have been a staple of laboratory-scale analytical procedures but are usually time/labor-intensive and often must be performed in non-continuous batches [10]. On-chip concentration methods have been developed to overcome these limitations. Some on-chip methods take advantage of the electrokinetic properties of analytes to concentrate them using velocity differences or focusing effects [10]. However, these methods (e.g., field-amplified stacking, ion concentration polarization, isotachophoresis, or isoelectric focusing) require an applied electric field, often using high voltages; they are generally limited to concentrating charged analytes, such as ions and proteins; and conditions for concentration are highly sensitive to flowrate and to sample composition [10,11]. Other on-chip concentration methods rely on surface-binding effects, such as immunoprecipitation. These methodologies are inherently not real-time, as they require constant cycling between binding and resolubilization of the analyte of interest. Also, many preconcentration methods provide difficult-to-quantify increases in concentration that make it challenging to correlate the measured concentration to the original concentration in the raw sample [10]. There is, therefore, a clear need for continuous, quantifiable, and simple preconcentration methods that work across a wide range of analyte classes (e.g., ions, molecules, proteins, etc.) and solutions.

Concentration merely is *solute* per *solvent*. By focusing on reducing the amount of *solvent* (usually water), broad-spectrum solutions become possible. Semi-permeable membranes are well suited for this approach [12]. When suitably selected, a semi-permeable membrane can allow the passage of water while leaving analytes of interest in the channel. The movement of water can be driven by either a pressure (reverse osmosis) or an osmolarity (forward osmosis) gradient. Reverse osmosis requires active, pneumatic control of high pressures and is thus innately more difficult to miniaturize down to an on-chip device. On the other hand, forward osmosis requires no external peripherals. However, previous attempts to use forward osmosis for preconcentration were not continuous, quantifiable, or simple [12,13].

**Fig 1:**
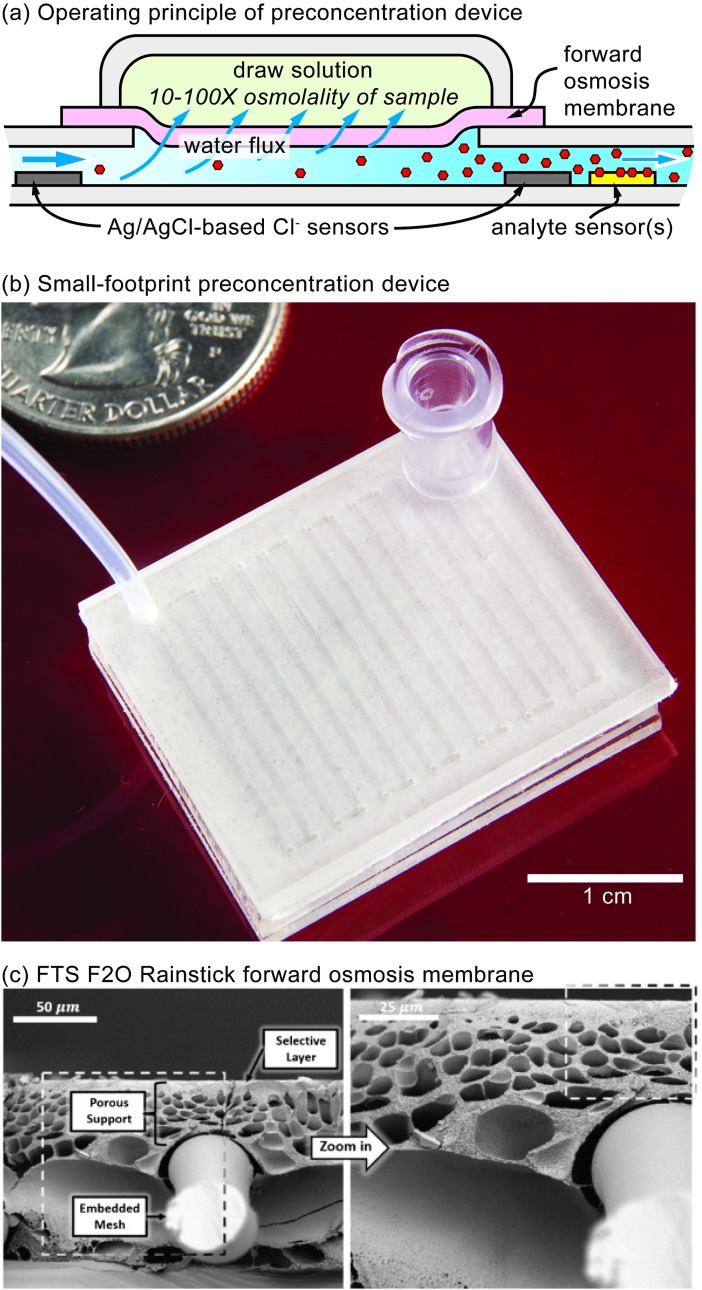
Principle of preconcentration device and proposed device components. (**a**) Cross-sectional diagram demonstrating the operating principle of the microfluidic preconcentration device. (**b**) A miniaturized preconcentration device designed with form-factor and manufacturability in mind, produced by ALine Microfluidics. (**c**) SEM image of the FTS F2O Rainstick forward osmosis membrane, *reproduced* [14].

In this paper, we demonstrate the first membrane-based, microfluidic preconcentration device that is continuous, quantifiable, simple, and capable of working with any analyte. A cross-section of the microfluidic device is shown in Fig 1A. A sample containing a dilute concentration of an analyte of interest flows into a microfluidic channel. The top of the channel is laminated with a semi-permeable membrane that rejects the analyte. A draw solution reservoir with a pre-determined high osmolarity sits on top of the membrane. Often, the sample will be a biofluid where the osmolarity is known to be within a tight range. The osmolarity gradient between the draw solution reservoir and sample solution will draw water into the draw solution reservoir, concentrating the sample until equilibrium is reached. Therefore, as long as the flowrate of the sample is below a critical threshold so that the sample osmolarity can equilibrate with the draw solution osmolarity, then the increase in concentration will be quantifiable. Furthermore, if the sample osmolarity is unknown or highly variable, placing Ag/AgCl-based Cl^-^ sensors before and after the concentrating module can be used to potentiometrically quantify the amount of preconcentration. In either setup, sensors can then be placed downstream to detect the analytes of interest. Fig 1B shows a working device with a small footprint and ready-for-manufacture design implemented by ALine Inc. Impressively, with <1 cm^2^ of exposed membrane area, complete 10-100x preconcentration occurs within mere minutes.

We also evaluated the performance of multiple combinations of membranes and draw molecules to optimize for our device. While a few combinations have been studied [15–18], no extensive cross-comparison has been performed. This paper is the first to perform a systematic optimization of the performance of several classes of membranes (i.e., forward osmosis, nanofiltration, and dialysis) in combination with a panel of differently-sized draw molecules (i.e., a salt, a sugar, and a polyelectrolyte).

Next, we built a proof-of-concept preconcentration device with sensors throughout its length. We developed a numerical model similar to that described by Phuntsho et. al [19]. We then conducted a set of characterization experiments and fit our model to the results. Also, we examined our selected membrane’s ability to concentrate two biologically relevant molecules: glucose and bovine serum albumin (BSA). Specifically, in the case of BSA, a traditionally sticky molecule, we investigated the membrane’s resistance to fouling.

## Materials and methods

### Membrane and draw molecule material selection

Both the membrane and draw molecule are critical components that require careful consideration. The membrane must reject the analytes of interest and the draw molecule while allowing water to flow through at reasonable rates. The size and chemistry of a membrane’s pores determine the threshold of molecular sizes and types that will be rejected, and the density and length of these pores determine the resistance to water. The draw molecule must be large enough to be rejected by the selected membrane but small enough to have high solubility in water (osmolarity) and low viscosity [15].

We chose membranes from three different classes—forward osmosis, nanofiltration, and dialysis—representing different pore sizes, porosities, constructions, and materials. All membranes were hydrophilic as this best supports water flux [20]. The forward osmosis membrane (FTS Rainstick) has pores with a diameter of just 0.7 nm and is the only membrane selected that rejects 97% of NaCl, as stated by the manufacturer. This membrane is designed to support high water flux and to resist fouling [21–24]. Several polyamide, nanofiltration membranes (Synder Filtration) with various molecule weight cutoff (MWCO) ranges were also selected: NFS (100–250 Da MWCO, 50% NaCl rejection), NFX (150–300 Da MWCO, 40% NaCl rejection), NFW (300–500 Da MWCO, 20% NaCl rejection), and NFG (600–800 Da MWCO, 10% NaCl rejection). Finally, several cellulose-ester, dialysis membranes (Biotech, Spectrum Labs) with the following MWCO ranges were selected: 0.1–0.5 kDa, 0.5–1.0 kDa, and 3.5–5.0 kDa.

We chose draw molecules that have been previously demonstrated in forward osmosis systems: a salt (NaCl) [15], a sugar (sucrose) [17], and a polyelectrolyte (polyethylenimine) [16]. If a membrane fails to reject Na^+^ and Cl^-^ (23 Da, 35 Da), then larger sucrose (342 Da) or very large polyelectrolytes like polyethylenimine (600 Da to 60 kDa) may be required by membranes with larger pores. While both NaCl and sucrose are similarly soluble in water (up to roughly 6 M), NaCl is advantageous, as it is much less viscous at these higher concentrations. Each subunit of a polyelectrolyte contributes to its osmolarity, allowing it to produce a large osmotic pressure while being easily rejected by most membranes. However, like sucrose, polyethylenimine is extremely viscous at the high concentrations needed to concentrate effectively [15,16]. Branched polyethylenimines were used with both low (M_n_ ~600 Da; P/N: 408719, Sigma-Aldrich) and high (M_n_ ~60 kDa; P/N: 181978, Sigma-Aldrich) molecular weights.

Additionally, membranes are stored either dry or wet, but once wet, a membrane should not be allowed to dry out, or else its properties may change. The forward osmosis and nanofiltration membranes are stored dry, but the dialysis membranes are pre-wetted. While not an issue for our membrane and draw molecule characterization experiments, pre-wetted membranes may be difficult to integrate into a device.

### Membrane and draw molecule evaluation

**Fig 2:**
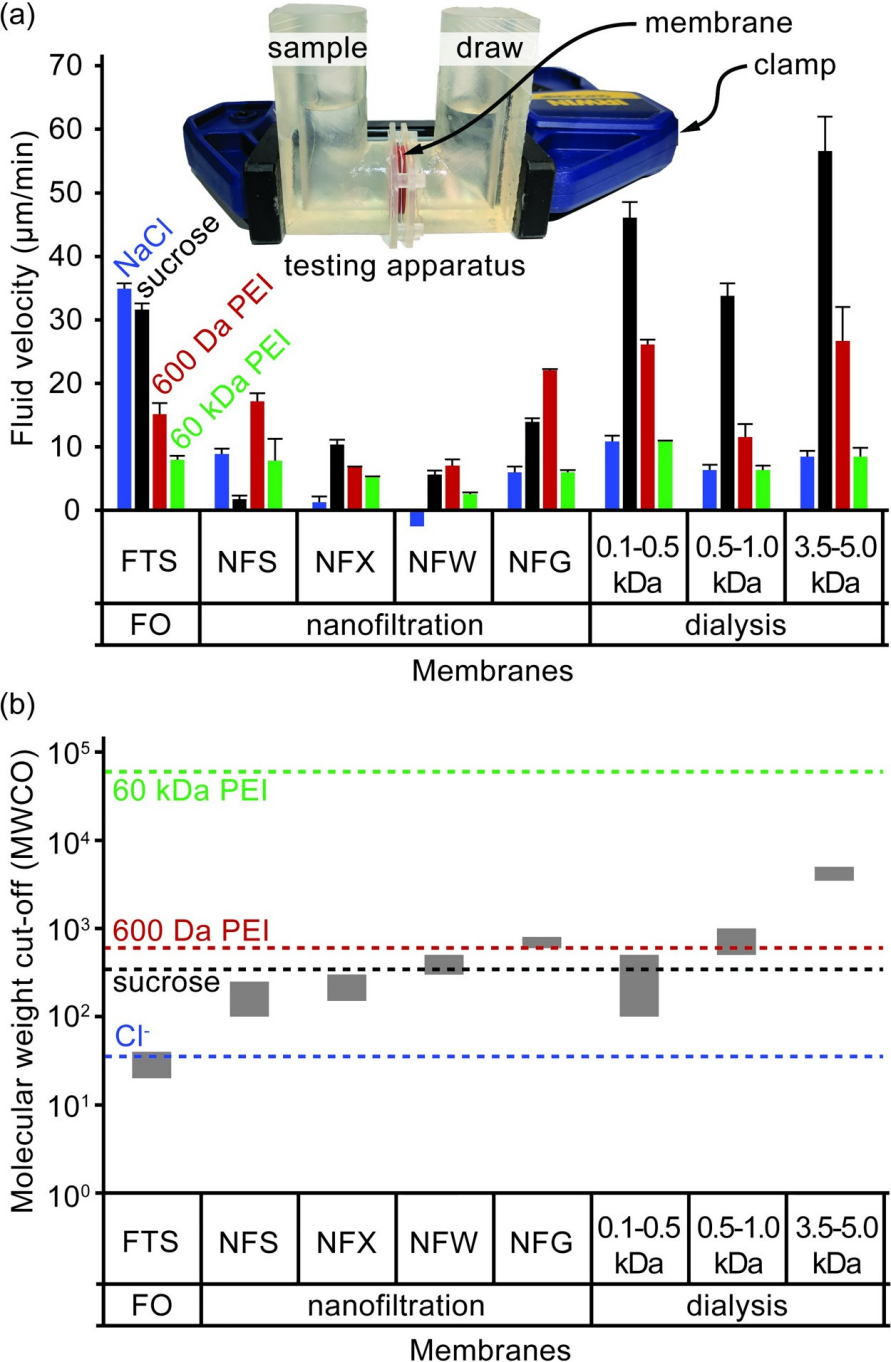
Testing results of membrane and draw molecule performance. (**a**) The forward osmosis performance of various combinations of membranes and draw molecules. Performance metric is the average water flux over a 3 h period. Sample is 10 mL deionized water; draw osmolarity is 1 OsM. Overlay image depicts testing apparatus. (**b**) The MWCOs of each membrane and MW of each draw molecule is plotted together on the same graph to give insights on the size compatibility of each combination. Three membrane classes are represented: forward osmosis (FTS), nanofiltration (NFS, NFX, NFW, and NFG), and dialysis (0.1–0.5 kDa, 0.5–1.0 kDa, and 3.5–5.0 kDa). Draw molecules are NaCl, sucrose, and both low and high MW branched polyethylenimine (PEI).

The performance of each membrane and draw molecule combination is evaluated using water flux. Water flux is measured using a testing apparatus that clamps a membrane between two interlocking reservoirs (Fig 2A inset). The reservoirs are printed using a high-resolution, stereolithographic 3D printer (Form 2, Formlabs) in clear photopolymer resin (FLGPCL02, Formlabs). Each reservoir holds roughly 10 mL of fluid and makes a fluid-tight seal against the membrane using an O-ring, leaving a 2.5 cm^2^ area of the membrane exposed for fluid transport. Membranes are wetted with deionized water for several minutes before being clamped within the testing apparatus. One reservoir is filled with 10 mL of deionized water. Then, the apparatus is weighed on an analytical balance. To begin the test, 10 mL of a draw solution are added to the other reservoir. The osmolarity of the draw solution was set to a constant, 1 OsM. However, the draw molecules being tested have different degrees of dissociation and so contribute differently to the osmolarity. NaCl dissociates into two ions, sucrose is nonionic, and polyethylenimine monomers dissociate into roughly two ions at pH 6.5. Therefore, the draw concentrations for NaCl, sucrose, and polyethylenimine were set to 0.5 M, 1 M, and 0.5 M, respectively. The apparatus is sealed using paraffin film to prevent any possible evaporative effects. After three hours, the draw solution is aspirated from its reservoir, and the apparatus is weighed again. Water flux is calculated as the difference in measured weights divided by both the duration and exposed membrane surface area. This procedure was performed in triplicate for all combinations of membranes and draw molecules.

### Preconcentration device construction

The traditional soft lithography techniques for producing microfluidic devices are quickly being replaced by even more rapid prototyping techniques [25–27]. We took advantage of the well-documented, design-cut-assemble approach [27] to produce our device layer-by-layer—a rigid support layer, a microfluidic channel patterned from double-sided tape, the selected semi-permeable membrane, and a draw solution reservoir. The entire device is built up from the rigid support layer. This layer is cut from a clear acrylic sheet (3 mm-thick, McMaster-Carr) using a laser cutter. Ag/AgCl ink (P/N: Cl-4001, Engineered Conductive Materials) is screen printed onto the acrylic support layer to provide six equally-spaced chloride-ion sensors throughout the length of the device. A straight microfluiedic channel (150 mm long; 0.5 mm wide) is laser cut out of double-sided, microfluidic tape (90 um thick, 3M 9965). The patterned double-sided tape is aligned with the inlet and outlet ports of the acrylic sheet and then laminated using a heated-roll laminator. Similarly, the membrane (FTS Rainstick) is laminated on top of the tape. Strips of the same microfluidic tape are laminated across the inlet and outlet ports to ensure that only the straight channel is exposed to the draw solution. The draw solution reservoir must hold several milliliters of draw solution (NaCl), and so a perimeter wall is cut out of acrylic and sealed to the top of the membrane with hot-melt adhesive. Evaporation can be prevented by covering the reservoir with paraffin film, plastic wrap, or another sheet of acrylic. Once all layers are assembled, ~20 cm lengths of polyetheretherketone (PEEK) tubing (0.03” ID, 1/16” OD) are pressed into the inlet and outlet ports that were cut on the acrylic support layer and then secured in place with UV curable epoxy (Loctite 352).

### Concentration profiles at various input flowrates

A device is clamped to a stand so that the draw solution reservoir is facing up. The PEEK tubing of the inlet is attached to an empty, 1 mL glass syringe (Hamilton 1000 series) via a compression fitting, and the PEEK tubing of the outlet rests in a beaker containing 5 mM NaCl and a double-junction Ag/AgCl reference electrode (P/N Z113107, Sigma-Aldrich). Only for experimental characterization (not needed in practice), the draw solution reservoir is initially filled with the same 5 mM NaCl so that the device channel can be filled without any water flux. A syringe press (KD Scientific Legato 111) withdraws the glass syringe at 20 μL/min until the 5 mM NaCl from the beaker is drawn through the channel and into the syringe. This withdrawal phase is critical to filling the channel without air bubbles.

The syringe is then removed from the syringe press. The syringe is disconnected from the inlet tubing by submerging the syringe and tubing in a large beaker of deionized water. The tubing is left submerged while the syringe is fully loaded with 5 mM NaCl. The syringe is primed, submerged, and then reattached to the tubing. This tedious, submersion process is critical to ensure that no air bubbles enter the channel. The syringe press infuses the 5 mM NaCl into the channel at 20 μL/min. The six screen-printed Ag/AgCl electrodes within the channel have an electrochemical potential that correlates to chloride ion concentration of the sample directly above the electrode when measured against the reference electrode in the outlet beaker. The six electrodes and the reference electrode are connected to a multi-channel, precision, high input impedance (10^13^ Ω) electrode interface (EMF6, Lawson Labs). The electrical potential of each of the six electrodes is recorded. This process is repeated for 50 mM and 500 mM solutions to produce a three-point calibration curve for each electrode. Between each test, the draw solution is aspirated, rinsed with the new solution, then aspirated again before refilling.

**Fig 3:**
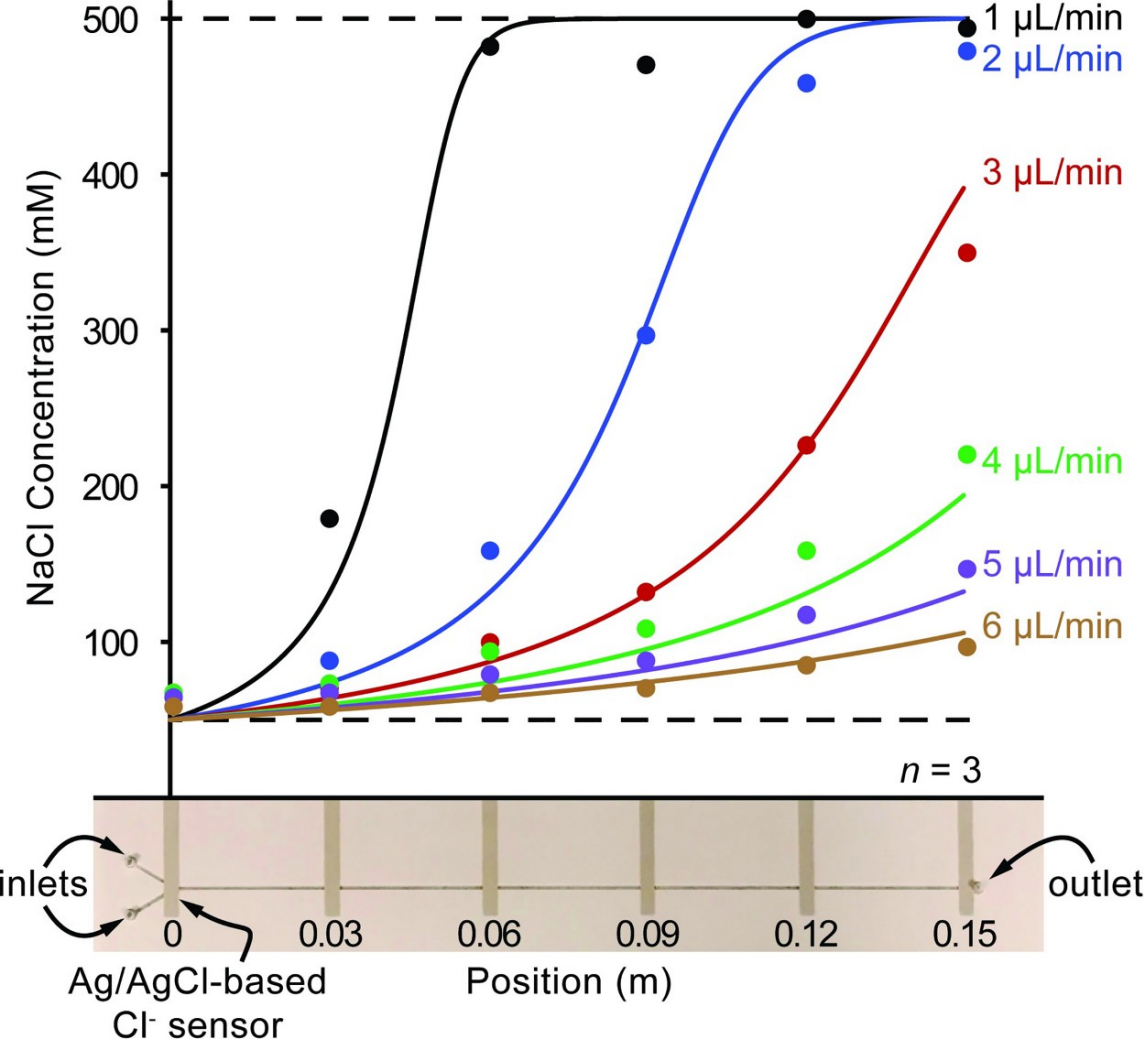
Concentration profiles of NaCl at discrete positions throughout straight-channel preconcentration devices operating at various input flowrates. The sample solution is 50 mM NaCl, and the draw solution is 500 mM NaCl which should concentrate samples by 10x. Concentrations are predicted by the *in-silico* model and plotted as curves for each flowrate, and the discreet chloride ion concentration measurements from the Ag/AgCl electrodes are plotted as points. A picture of an actual preconcentration device is depicted below the graph. The location of the screen-printed electrodes are shown below the x-axis. The volume of the feed is based on the channel dimensions: 150 mm x 0.5 mm x 90 μm = 6.75 μL. The volume of the draw is based on the draw reservoir dimensions: 160 mm x 35 mm x 5 mm = 28 mL. The channel volume is just 0.02% that of the draw volume. Therefore, the dilution effects are negligible.

A profile of the concentration as a function of position in the channel can then be determined for various input flowrates (Fig 3). In this particular experiment, the device is configured to concentrate a 50 mM NaCl sample solution by 10x. The draw solution was replaced with 500 mM NaCl to provide the 10x osmolarity gradient, and the syringe was replaced with 50 mM NaCl and then infused at various input flowrates (1 μL/min to 6 μL/min). The concentration at each of the six electrode positions was recorded after stabilizing.

The calibration procedure is performed both before and after generating the profile to account for electrode drift. Reported concentration values are corrected to account for this drift and averaged from three independent runs each using a separate channel.

### Biological relevance

**Fig 4:**
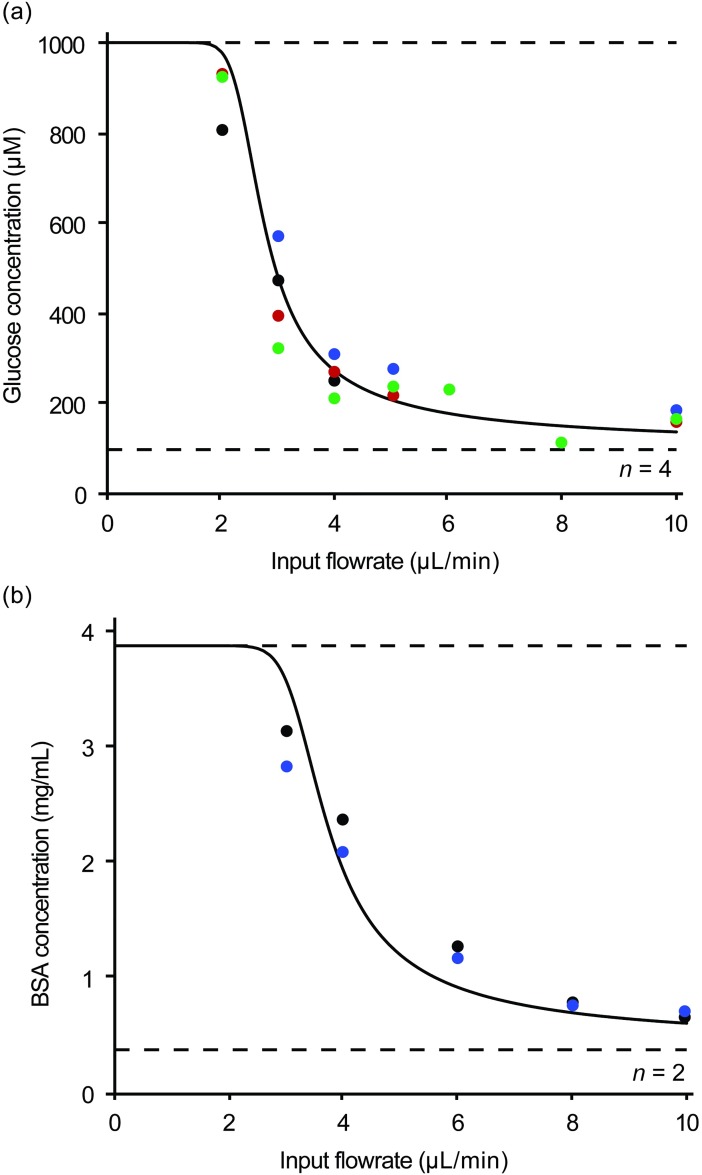
Predicted and recorded concentration profiles of biologically-relevant analytes at varying flowrates. The concentrations at various input flowrates are shown for two biologically-relevant analytes: glucose and BSA. Concentrations are predicted by the *in-silico* model and plotted for each analyte.

To test if the device is capable of concentrating biologically relevant molecules without fouling, we selected two molecules: glucose, a small sugar, and bovine serum albumin (BSA), a large sticky protein. A sample containing 100 μM glucose in 0.1x phosphate-buffered saline (PBS) is concentrated by 10x using a 1x PBS draw solution, and a sample containing 0.39 mg/mL BSA in 1x PBS is concentrated by 10x using a 10x PBS draw solution. A device is prepared and pre-filled with the respective sample using the withdraw-based filling method as described above. Once the device is filled, the syringe is removed and filled with the respective sample using the submersion technique described above. Then, the syringe pump infuses the sample at various flowrates into the device. Concentrated samples from the outlet are collected and analyzed (Fig 4A).

Glucose concentrations are determined using electrochemical glucose test strips designed for diabetes management. These test strips can be hooked up to a lab-grade potentiostat (Gamry Instruments Reference 600+) rather than a glucometer to increase the precision of the reading [28]. Test strips are preferred over traditional assay techniques, because they require less than 1 μL of sample and provide results in less than a minute. BSA concentrations are determined by absorption at 280 nm using a UV-vis spectrophotometer (NanoDrop One Microvolume, Thermo Scientific). This technique also requires less than 1 μL of sample and provides instantaneous results. Glucose concentration was replicated in four separate channels, and BSA concentration was replicated in two separate channels (Fig 4B).

### Predictive in-silico model

We have developed an in-silico model that accurately predicts the behavior of the preconcentration device. The model simulates a sample solution (with a given osmotic concentration and input flowrate) flowing through a microfluidic channel (with a given length, width, and height). The sample solution is separated from a draw solution (with a given osmotic concentration) by a membrane (with a given resistance). The model, written in C++ (see online supplementary file), performs the following on each discrete clock cycle, or tick:

1. **A block of fluid is added to the channel inlet**.
  (Volumeofblockadded)=(Inputflowrate)(Durationoftick)
2. **Loop through each block and remove volume lost through the membrane due to osmosis.**
  $$(Flow\;rate\;through\;membrane)=\frac{\left(\left(Draw\;solution\;conc.)-(Block\;conc.\right))(iRT)(Membrane\;area\right)}{\left(Membrane\;Resistance\right)}$$
  (Newvolumeofblock)=(Oldvolumeofblock)−(Flowratethroughmembrane)(Durationoftick)
3. **If blocks have reached the outlet of the device, the model is finished. If not, begin next tick**.

The model outputs the flowrate and concentration at every block along the channel as well as the time needed to reach the outlet. Experimental outlet concentration data collected at various flowrates can be used to determine the membrane resistance. For simplicity, internal (ICP) and external (ECP) concentration polarization are not considered in our model. In forward osmosis, ECP is negligible, and ICP only changes when the membrane, draw molecule, or driving osmolarity gradient is changed. Since we optimized our membrane and draw molecule selection, they will not change. As a result, we can consider our membrane resistance term to have the added resistance of ICP “baked in” for a set osmolarity gradient. Therefore, as long as we re-fit the model whenever the osmolarity gradient changes, it can be used as a useful, predictive tool. The trend lines shown in the figures represent the model output for those experiments.

**Fig 5:**
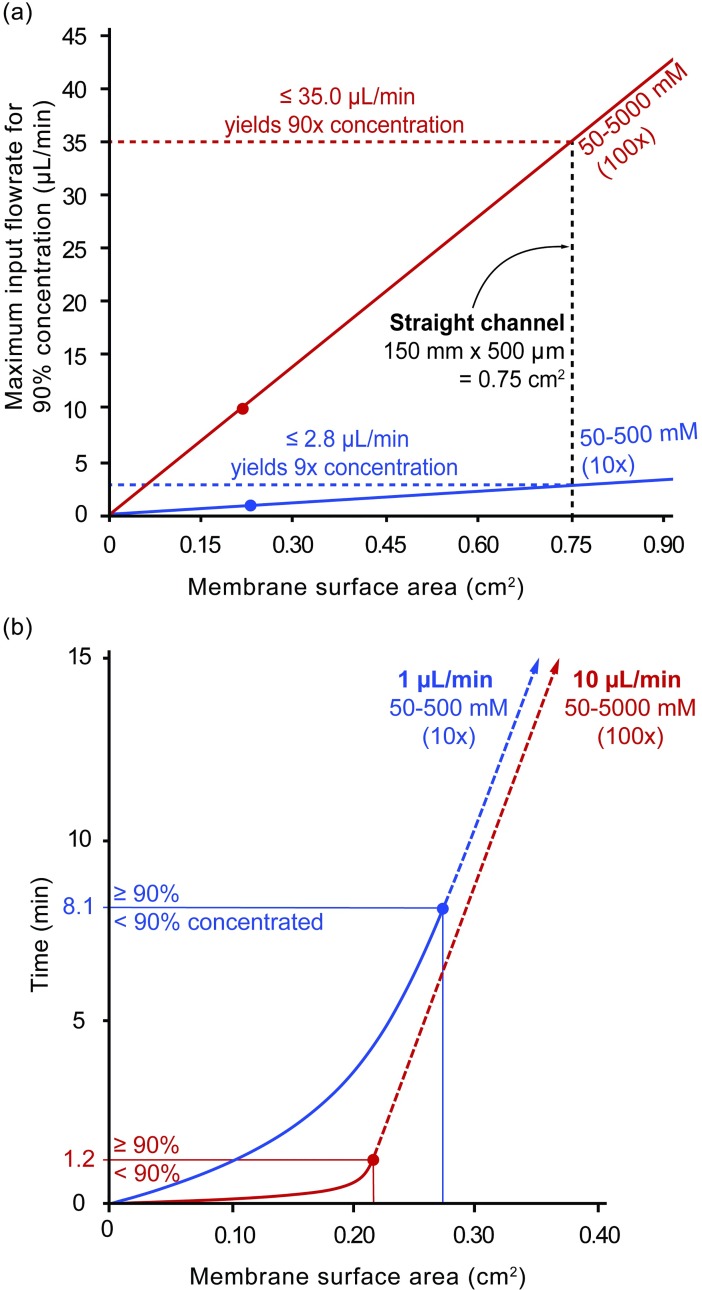
*In-silico* model predictions for design constraints and considerations. (**a**) The *in-silico* model predicts a linear proportionality between membrane surface area (cm^2^) and the maximum input flowrate that is capable of concentrating to 90%. (**b**) The time (min) needed to for the sample to travel over a given membrane surface area (cm^2^) as predicted by the *in-silico* model.

The model was run using both a 10x (50–500 mM concentration gradient) and a 100x (50–5000 mM concentration gradient). The input width and height were as described for the straight channel. The input length was swept from 0 to 150 mm, meaning that the membrane surface area ranged from 0 to 0.75 cm^2^. At each of these lengths, the input flow rate was increased until less than 90% concentration was achieved. The outputs of the swept length and flow rate runs for the two different concentrations (10x and 100x) yielded the relationships depicted in Fig 5.

## Results and discussion

### Forward osmosis performance factors

Forward osmosis performance can be affected by concentration polarization, a buildup of concentration gradients internal or external to a membrane [29,30]. This reduces the effective osmotic gradient across the active layer of the membrane thereby reducing the water flux. Internal concentration polarization (ICP) results from the fact that most membranes are asymmetric, having a dense, active layer and a porous, support layer (S1 Fig). Membranes can be oriented with the active layer facing either the feed or draw solution. When the active layer faces the feed solution, draw molecules from the bulk solution must diffuse into the support layer against the flow of incoming water. As a result, the draw solution is diluted at the interface between the active and support layer. Because the concentration of the draw solution is much larger than the feed solution, a dilution on the draw side dramatically reduces the osmotic gradient, and so the water flux is diminished. The effects of ICP are exacerbated when the thickness of the support layer increases, the porosity of the support layer decreases, water flux increases, or diffusivity of the draw molecule decreases. ICP can largely be avoided by flipping the orientation of the membrane. However, since the active layer is less prone to fouling than the support layer, we orient the membranes such that active layer always faces the sample. External concentration polarization (ECP) occurs at the surface of the membrane due to water being removed from the feed side causing a local region of concentration and water fluxing to the draw side causing a local region of dilution. However, the water fluxes involved in forward osmosis are so low that ICP dominates, and ECP can be ignored.

### Membrane and draw molecule performance evaluation

The water flux for each membrane and draw molecule combination is reported in Fig 2A using a constant 1 OsM gradient. The NaCl draw solution is most effective when used with the salt-rejecting FTS membrane. However, with all other membranes, the NaCl draw solution is one of the poorest performers due to these membranes’ poor rejection of NaCl. Based on their molecule weight cutoffs (MWCOs), the FTS, NFS, and NFX membranes should reject sucrose, the NFW and the 0.1–0.5 kDa dialysis membranes may partially reject it, and all the rest should largely allow it to pass (Fig 2B). The sucrose draw solution has a slightly lower water flux compared to the NaCl draw solution across the FTS membrane even though the osmolality of the sucrose solution is roughly 30% greater than NaCl due to the larger viscosity. It is possible that this is accounted to an increased internal concentration polarization (ICP) as a result of the larger viscosity of the sucrose draw. The sucrose draw solution produces the lowest water flux through the NFS membrane, suggesting that the NFS membrane may not be rejecting sucrose as expected or sucrose is fouling this membrane. The sucrose draw solution also produces low water fluxes in the other nanofiltration membranes (NFX, NFW, and NFG) compared to the FTS and dialysis membranes. The dialysis membranes have the largest pore sizes and exhibited large water fluxes when combined with a sucrose draw. Surprisingly, regardless of the draw solution, the water flux does not increase as expected as pore size increases within the nanofiltration (NFS, NFX, NFW, or NFG) or the dialysis (0.1–0.5 kDa MWCO, 0.5–1 kDa MWCO, or 3.5–5.0 kDa MWCO) membrane classes. The low molecular weight polyethylenimine should be rejected by the NFG nanofiltration, 0.5–1.0 kDa MWCO dialysis, and 3.5–5.0 kDa MWCO dialysis membranes, and the high molecular weight polyethylenimine should be rejected by all membranes. In all membranes, the low molecular weight polyethylenimine produces higher water fluxes than the high molecular weight polyethylenimine, suggesting that all membranes likely reject these bulky, branched polyelectrolytes and that the higher viscosity of the high molecular weight polyethylenimine increases ICP. In summary, dialysis membranes generally produce higher maximum water fluxes than forward osmosis membranes, and nanofiltration membranes had the lowest maximum water fluxes.

A sucrose draw solution combined with the largest dialysis membrane (3.5–5.0 kDa MWCO) produces the most water flux of any membrane/draw molecule combination. However, the large MWCO of this membrane limits the size of analytes that can be concentrated. For most proteins, this size limit is acceptable, but analytes such as metabolites, signaling molecules, or hormones are much too small. An NaCl solution combined with the tightest membrane (FTS) produces a comparably high water flux without the same size limitation for potential analytes. While sucrose could also be used with the FTS membrane, NaCl is particularly advantageous because of its lower viscosity and ubiquity in biofluids. Therefore, NaCl and the FTS membrane were determined to be the preferred approach for the remainder of experimentation. We initially speculated that the FTS membrane would excel, because it was specifically designed as a pouch for quickly generating potable water by forward osmosis even in a muddy puddle (e.g., must block small molecule biotoxins, etc.).

### Preconcentration device operating regimes

To aid our description of device function, we define two distinct operating regimes: *preconcentration-limited* and *transport-limited*. During the *preconcentration-limited regime*, the sample undergoes preconcentration throughout the channel. At higher flowrates, the sample never reaches osmotic equilibrium, resulting in incomplete concentration (Figs 3 and 4). Interestingly, while the maximum osmotic gradient exists at the beginning of the channel, not much preconcentration occurs there. Rather, as the sample concentrates, the flowrate decreases and allows more time for water to be removed, increasing the rate of preconcentration further down the channel. However, as the sample osmolarity nears the draw solution osmolarity, the driving osmotic gradient decreases, and the rate of concentration again slows. Finally, the sample osmolarity equilibrates with the draw solution osmolarity, and preconcentration completes. The sample then exits at a fraction the input flowrate (1/10 for 10x, 1/100 for 100x, etc.). These slow, fast, then slow preconcentration rates result in the visually obvious sigmoidal profiles seen in Fig 3. However, at low flowrates, samples do reach osmotic equilibrium. The *transport-limited regime* occurs after the sample has fully concentrated, reached its lowest flowrate, and then must merely continue to travel at this low flowrate until reaching the outlet.

### Concentration profile experiments

To examine the concentration profile for each input flowrate, the chloride ion concentration was measured at discrete locations throughout the channel. For each input flowrate, Fig 3 plots both measured (points) and predicted (curves) concentration for each position along the channel. The predicted concentrations were generated with the *in-silico* model as described above. The membrane resistance calculated by the *in-silico* model is 3e12 Pa-s/m, and the standard errors of the regression for each flowrate in ascending order are as follows: 4.7%, 3.9%, 3.7%, 3.7%, 2.1%, and 1.1%.

As shown in Fig 3, increasing the input flowrate also increases the length of channel needed to fully concentrate the input sample. This is because both the resistance of the membrane and pressure gradient across the membrane are largely constant, and so the flux is also constant. Therefore, as the input flow rate increases the solution spends less time at each unit length of the channel, meaning less water is removed. The extent of concentration at each unit length of the channel is directly proportional to the input flow rate. For example, an input flowrate of 1 μL/min reaches 90% concentration (400mM) at roughly 0.05 m within the channel, while an input flowrate of 2 μL/min does not reach the same concentration until 0.1 m.

### Biological relevance experiments

The outlet concentrations at various input flowrates of both glucose and BSA also follow the sigmoidal profile predicted by the *in-silico* model (Fig 4). Based on the *in-silico* model, input flowrates less than 1.9 μL/min for glucose and 2.5 μL/min for BSA will allow samples to be concentrated to >99% of the expected 10x. Experimentally, an input flowrate of glucose at 2 μL/min and BSA at 3 μL/min yields 97% and 77% completion, respectively. The membrane resistance calculated by the *in-silico* model is 1e12 Pa-s/m for glucose and 7.6e12 Pa-s/m for BSA, suggesting slight membrane fouling by BSA. The standard error of the regression is 3.4% for glucose and 4.7% for BSA.

BSA may somewhat adhere to the channel walls, reducing recovery at the outlet thus causing a lower concentration to be measured. The lower input flowrates move slower through the channel and concentrate to a greater extent, causing further slowing. As a result, these lower flowrates likely experience greater BSA adhesion to the channel walls. This could explain the model’s difficulty in fitting this data. Sample analyte adhesion to the channel walls is a potential problem, but existing pre-treatment techniques [31] could help but were not examined in this work. It should be noted that neither glucose nor BSA was experimentally shown to reach 100% of the expected concentration, because neither was run at flowrates less than 2 μL/min and 3 μL/min, respectively. The time needed to collect an adequate sample at these lower flowrates would be prohibitively long without modifying device parameters.

### Design considerations

Based on the *in-silico* model, membrane surface area is proportional to the maximum input flowrate that is capable of concentrating to 90%. In Fig 5A, this relationship is shown for two concentration gradients: 10x (50–500 mM) and 100x (50–5000 mM). The slope of the 100x gradient (47 μL/min per cm^2^) is roughly ten times greater than that of the 10x gradient (3.7 μL/min per cm^2^). While flowing at a flowrate less than the threshold flowrate will yield concentrations greater than 90%, the time spent in the channel will increase. For example, preconcentration of samples flowing at 10 μL/min by 90x and 1 μL/min by 9x requires a membrane surface area of just 0.22 cm^2^ and 0.27 cm^2^ and takes only 1.2 mins and 8.1 mins, respectively (Fig 5B). However, by using a similar membrane surface area as our straight channel (0.75 cm^2^), it would take both almost an hour to reach the outlet. The solid lines in Fig 5B represent the short *preconcentration-limited* regime while the dashed lines represent the long *transport-limited* regime. Matching the membrane surface area to the range of flowrates that a device will experience during operation is critical to minimize the latency.

## Conclusions

We have demonstrated that forward osmosis can be used to preconcentrate samples within a microfluidic device both continuously and in real-time. Our selected membrane showed good resistance to fouling from the biologically-relevant solutions tested. In addition, we developed an *in-silico* model that predicts device functionality based on design parameters, providing an engineering toolkit for future designs. We used this toolkit to optimize our device, allowing us to produce inexpensive, ready-for-manufacturing prototypes that preconcentrate 10-100X within minutes.

Preconcentration is just one of many other preprocessing steps such as desalting, buffering pH, removing interferents, and delivering reagents. For this reason, this preconcentration method may be combined with other steps to provide a complete solution for specific sensing applications. For example, concentrating analytes in a biofluid using a salt-rejecting membrane will also increase the salt concentration. Some chemical biosensors have altered performance when the salt concentration is outside of physiological ranges. For unbuffered fluids, pH changes could also be a challenge for some sensors. Fortunately, microfluidic approaches have been developed for modulating salt concentration [32], and methods of buffering pH such as the addition of a buffer can be easily scaled down to a microfluidic device. Now that we have demonstrated a microfluidic solution for continuous, quantifiable, and simple preconcentration, next-generation lab-on-a-chip devices targeting high-impact applications only need to focus on the integration of specific sensing modalities, and if needed, these modular sample preprocessing steps.

## Supporting information

S1 Fig Schematic representation of (**a**) dilutive internal concentration polarization (ICP) and (**b**) concentrative internal concentration polarization (ICP), *reproduced* [29].
(TIFF)
