# Supplementary figures and images for: Continuous, quantifiable, and simple osmotic preconcentration and sensing within microfluidic devices
Source: PLoS One. 2019 Jan 16;14(1):e0210286. doi: 10.1371/journal.pone.0210286 (PMC6334995; doi:10.1371/journal.pone.0210286)

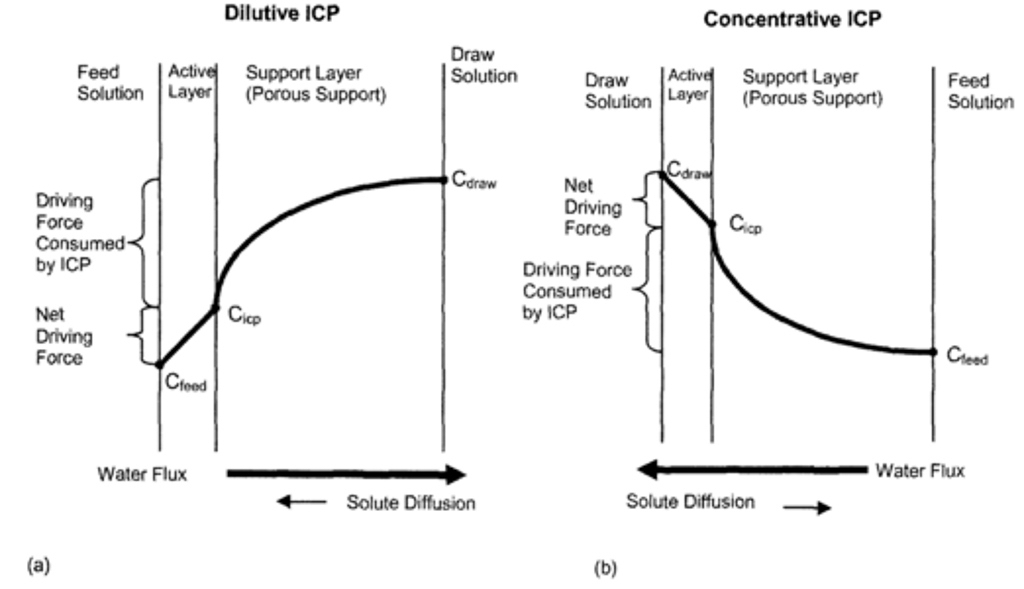

Supplement: S1 Fig — Schematic representation of (a) dilutive internal concentration polarization (ICP) and (b) concentrative internal concentration polarization (ICP), reproduced [29]. (TIFF) [file pone.0210286.s001.tiff]
